# Beneficial effects of pumpkin seed oil as a topical hair growth promoting agent in a mice model

**DOI:** 10.22038/AJP.2019.13463

**Published:** 2019

**Authors:** Valiollah Hajhashemi, Parvin Rajabi, Mahdieh Mardani

**Affiliations:** 1 *Department of Pharmacology and Isfahan Pharmaceutical Sciences Research Center, School of Pharmacy and Pharmaceutical Sciences, Isfahan university of Medical Sciences, Isfahan, Iran*; 2 *Department of Pathology, School of Medicine, Isfahan University of Medical Sciences, Isfahan, Iran*

**Keywords:** Androgenic alopecia, Cucurbita pepo, Pumpkin seed oil

## Abstract

**Objective:**

Pumpkin (*Cucurbita pepo* L.) seed oil mainly consists of saturated and unsaturated fatty acids. Previously, it was reported that oral administration of pumpkin seed oil (PSO) improved hair growth in male pattern alopecia. This study aimed to evaluate hair promoting activity of topical PSO in an animal model.

**Materials and Methods:**

Male Swiss mice (25-30 g) were used. Dorsal hair of mice (2 x 2.5 cm) was gently removed. Groups were treated as follows: (A) Intact control (did not receive testosterone) (B) Testosterone solution only (5% w/v); (C) Testosterone (5%) + PSO (5%); (D) Testosterone (5%) + PSO (10%) (E) Testosterone (5%) + minoxidil (2%). Application of drugs (100 µl) was done for six days a week, for 3 weeks. Observational and microscopic examinations were performed and results of different groups were compared.

**Results:**

Topical application of testosterone significantly (p<0.01) prevented hair growth (compared with intact control). PSO (10%) increased hair growth score after 3 weeks and histopathological findings confirmed these results. After 3 weeks of treatment, the percentage of follicles in anagen phase was 95±4.6 and 44.4±15 for intact control and testosterone-only treated group, respectively. These percentages for PSO (10%) and minoxidil were 75±5.3 and 91.3±4.4, respectively and they could significantly (p<0.001) reverse the effects of testosterone.

**Conclusion:**

In conclusion, as topical application of PSO showed hair growth promotion, it might be regarded as a promising alternative for treatment of male pattern alopecia. Also, considering its composition, free fatty acids and minor components like phytoestrogens and vitamin E may have contributed to this effect.

## Introduction

Male pattern hair loss (MPHL) or androgenic alopecia (AGA), is the most common cause of hair loss in men. The incidence of MPHL varies among populations. Hair is an important feature of self-image and although hair loss does not have a direct impact on health but it can be distressing. Studies have shown men who suffer from MPHL are 75% less confident, especially when interacting with the opposite sex. Therefore, treatment of hair loss is an important issue (Wells et al., 1995).

Currently, only two drugs are approved by the FDA for the treatment of AGA. One is a potassium channel opener called minoxidil and the other drug that inhibits dihydrotestosterone synthesis is finasteride. These drugs should be consumed daily for long periods and are associated with several side effects (McClellan and Markham, 1999; Shin et al., 2007). Although androgens are the most important regulators of hair growth, some other hormones including thyroid hormones and glucocorticoids also affect human hair growth. The action of androgens in the hair follicles depends on their local bioavailability. Even if men with AGA have normal androgen levels in regular circulation, testosterone and dihydrotestosterone (DHT) are produced locally. All of the enzymes that are necessary for the androgenic metabolism in the skin, are available and can independently control follicle growth (Dutton et al., 2000; Poor et al., 2002; Stenn et al., 1993; Zouboulis, 2004). The purpose of the treatment is to prevent the process of miniaturization of follicles and, if possible, to return the process. 

In recent years, a variety of plant extracts or their bioactive components have been evaluated for their potential hair growth promoting properties. *Serenoa repens, Pygeum africanum, Urtica dioica, Camellia sinensis*, and polyphenols in green tea, exhibited 5*α*-reductase inhibitory effect and promoted hair growth (Lourith and Kanlayavattanakul, 2013; Shimizu et al., 2000). In a recent study conducted by Cho et al. (2014), it was reported that oral administration of 400 mg of pumpkin seed oil (PSO) for 24 weeks to men with androgenic baldness, increased hair growth. Pumpkin (*Cucurbita pepo* L.) is an annual climber plant and its seeds have considerable amount of oil with nutritional and medicinal importance (Adams et al., 2011). Moreover, pumpkin has antioxidant (Chen and Huang, 2018), anti-inflammatory (Yadav et al., 2010), antimicrobial (Hammer et al., 1999), cytoprotective (Shayesteh et al., 2017) and antidiabetic (Adams et al., 2011; Boaduo et al., 2014) effects. 

Pumpkin seed oil chiefly consists of saturated and unsaturated fatty acids, including myristic acid, palmitic acid, oleic acid, linoleic acid and linolenic acid. It also contains beta-sitosterol, which has inhibitory effects on 5α-reductase enzyme (Cabeza et al., 2003). Raynaud et al (2002) reported that linoleic acid also has inhibitory activity against 5α-reductase. Based on above-mentioned researches, this study was designed to evaluate hair growth promoting potential of topical PSO in a mice model of androgenetic alopecia. 

## Materials and Methods

**Drugs**

Testosterone propionate (Sigma, USA), formalin (Merck, Germany), pumpkin seed oil (PSO, Zarband, Iran), minoxidil (Pakdaroo, Iran) were used in this study.

**Animals**

Male Swiss mice (25-30 g) were purchased from animal house of School of Pharmacy, Isfahan University of Medical sciences, Isfahan, Iran. Six mice were housed in each cage and kept under 12 hr light/dark cycles at 24±2°C. Animals had free access to pellet diet and water. All experiments were performed according to guidelines for animal care issued by Isfahan University of Medical Sciences.

**Animal groups**

Animals were randomized in 5 groups of six to eight animals per group. Dorsal hair of mice in an area of 2 by 2.5 cm, was gently removed by a depilatory cream. Groups were treated as follows: (A) Intact control (did not receive testosterone) (B) Testosterone solution only (5%); (C) Testosterone (5%) + Pumpkin seed oil (PSO, 5%); (D) Testosterone (5%) + PSO (10%) and (E) Testosterone (5%) + minoxidil solution (2%). Mice in all groups, except group A, received testosterone (100 µl) topically once daily. Application of all drugs (100 µl) was done for six days a week for 3 weeks.

**Qualitative evaluation of hair growth**

Hair growth in each mouse was evaluated by visual observations and scored from 0 to 5 at 1^st^, 2^nd^ and 3^rd^ weeks of treatment. The scores (0= no growth, 1= up to 20% growth, 2= 20-39% growth, 3= 40-59% growth, 4= 60-79% growth and 5= 80-100% growth) were defined according to the method previously described by Kwon et al (2015).

 **Quantitative evaluation of hair growth**

At the end of the treatment period, animals were sacrificed and a 1 cm x 1 cm section of dorsal skin was cut and fixed in 10% formaldehyde solution. The fixed tissues were embedded in paraffin and then, appropriate slices were prepared for staining. The slices were stained with hematoxylin and eosin and analyzed using a light microscope. All samples were coded and the evaluation was done by a pathologist in a blinded way. Follicles in the anagen phase (active growth phase) and those in nonanagen phase (resting phase) were counted, and percentage of follicles in anagen phase was determined.

**Statistical analysis**

Mann Whitney U test was used for comparison of non-parametric data. One way analysis of variance (ANOVA) followed by Scheffe *post hoc* was used for parametric values using SPSS package (version 13.0). P values less than 0.05 were considered significant. 

## Results

**Effect of PSO on hair growth scores**

The results are summarized in Table 1. Hair growth score in testosterone-treated group was significantly (p<0.01) lower than that of no treatment group (intact control). Application of PSO could prevent the effect of testosterone on hair and this effect was significant (p<0.05 for 10% PSO). Minoxidil as the standard drug could also show a significant (p<0.01) hair growth promotion. 

**Table 1:** Effect of pumpkin seed oil on hair growth scores

**Groups**	**Weeks after treatment**		
	**1**	**2**	**3**
Control	1 (1-2.5)	3.5 (2-5)	4 (4-5)
Testosterone alone	1 (0.5-2)	2.5 (1-4)	2.5^##^ (1-4)
Pumpkin seed oil 5%	0.5 (0.5-1)	1 (0.5-1.5)	3 (2-3)
Pumpkin seed oil 10%	1 (0.5-5)	2 (1-5)	4^*^ (2-5)
Minoxidil 2%	1.5 (0.5-4)	3.5 (2.5-4.5)	4.5^**^ (4-5)

In each cell, the upper number is the median and the numbers in parenthesis shows the range. *p<0.05 and **p<0.01 compared to testosterone-only treated group. ##p<0.01 compared to control group (Mann Whitney U test was used for statistical analysis).

**Effect of PSO on cells in anagen phase**

**Figure 1:**
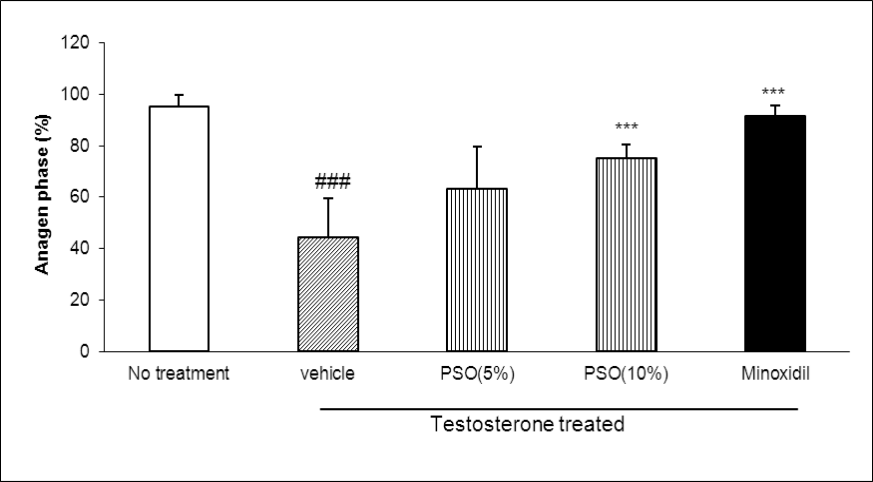
Effect of pumpkin seed oil on the percentage of hair follicles in anagen phase. PSO; pumpkin seed oil. ###p<0.001 compared to no treatment group. ***p<0.001 compared to testosterone-only treated group

**Figure 2:**
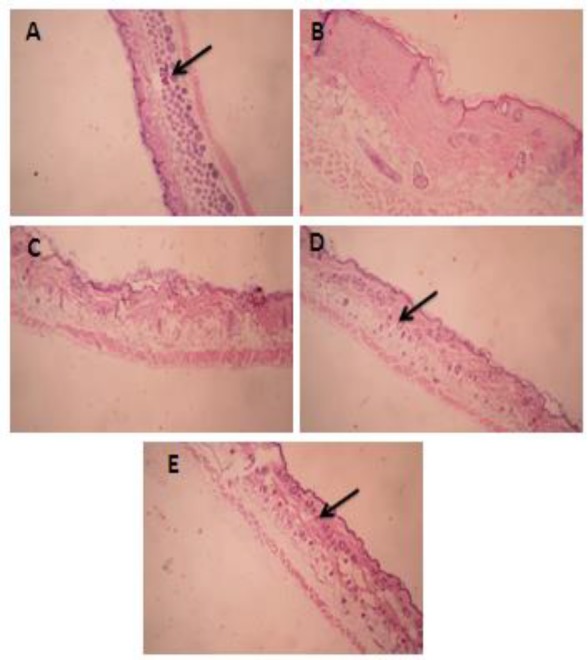
Pathological findings. 100 µl of testosterone solution (0.5% in alcohol) was applied on the shaved back skins of mice of all groups except group A (no treatment) one hour prior to application of drugs. Treatments continued once daily, 6 days a week for 3 weeks. At the end of the treatment period, skin samples were prepared, fixed in formalin and then, sectioned and stained with hematoxylin and eosin. A. No treatment; B. Testosterone alone; C. Testosterone+pumpkin seed oil (5%); D. Testosterone+pumpkin seed oil (10%); E. Testosterone+Minoxidil 2%. Arrows show the hair follicles. Magnification X40

According to light microscopy examinations, the percentage of follicles in anagen phase was determined for each treatment group (Figure 1 and 2). After 3 weeks, the percentage of follicles in anagen phase in intact control group, was 95±4.6. Local application of testosterone significantly (p<0.001) reduced the percentage of follicles in anagen phase. Pumpkin seed oil (10%) and minoxidil (2%) could significantly (p<0.001) reverse the effects of testosterone. 

## Discussion

In this study, topical administration of pumpkin seed oil (PSO) could reverse testosterone-induced hair growth retardation in mice and to the best of our knowledge, this is the first report on topical administration of PSO and hair growth. Previously, Cho et al (2014) in a double-blind trial demonstrated that oral administration of 400 mg of PSO per day for a period of 24 weeks, significantly increased hair count in comparison with placebo (40% in PSO-treated vs. 10% in placebo group). A drug like finasteride is used for both benign prostatic hyperplasia and male pattern baldness and in both cases, inhibition of 5*α*-reductase is involved. Interestingly, PSO has both of the above-noted effects. In a study conducted by Gossell-Williams (2006), it was reported that PSO inhibited testosterone-induced enlargement of the prostate in rats (Gossell-Williams et al., 2006) and human studies confirmed that PSO is an effective treatment for symptomatic BPH (Hong et al., 2009).

Based on our findings and previous reports, it may be concluded that PSO exerts its beneficial effects on hair follicle and prostate in a similar manner to finasteride. Generally, PSO reduces the androgenic activity of testosterone both in prostate (Gossell-Williams et al., 2006; Hong et al., 2009) and hair follicle (our findings) but whether the exact mechanism is mediated through inhibition of 5*α*-reductase or antagonistic effect at androgen receptors, is not clear and further investigations are needed. 

Some essential fatty acids, especially linoleic acid, are believed to inhibit 5-reductase (Raynaud et al., 2002) and researchers have attributed the hair growth activity of red ginseng oil (Truong et al., 2017) and rice bran (Choi et al., 2014) to linoleic acid. Similarly Shimizu et al reported that an acetone extract of *Boehmeria nipononivea* has hair regrowth activity and 5-reductase inhibitory effect and considering the chemical composition of the extract, they concluded that oleic acid and linolenic acid are responsible for the observed effects (Shimizu et al., 2000). Evaluation of the pure fatty acids confirmed the claim. Since the mentioned fatty acids compose a high percentage of PSO, they may contribute to our findings. According to the literature, PSO is a rich source of unsaturated fatty acids, especially oleic and linoleic acid, so that they make up about 70% of the total fatty acids (Murkovic and Pfannhauser, 2000) and therefore, it might contribute to hair growth observed in this study. Although linoleic acid is a possible contributor, the role of vitamin E and phytoestrogens (Camacho-Martinez, 2009) present in PSO on hair growth promotion, could not be neglected.

In conclusion, PSO showed hair growth promotion in a testosterone model and according to its composition, free fatty acids and other minor components like phytoestrogens and vitamin E contribute to the observed effects.
